# Construction of a prognostic model for hepatocellular carcinoma patients receiving transarterial chemoembolization treatment based on the Tumor Burden Score

**DOI:** 10.1186/s12885-024-12049-4

**Published:** 2024-03-06

**Authors:** Jiawei Lin, Jie Li, Yifan Kong, Junhui Yang, Yunjie Zhang, Guoqing Zhu, Zhijie Yu, Jinglin Xia

**Affiliations:** 1 Key Laboratory of Diagnosis and Treatment of Severe Hepato-Pancreatic Diseases of Zhejiang Province, The First Affiliated Hospital of Wenzhou Medical University, Wenzhou, China; 2 Department of Interventional Radiology, The First Affiliated Hospital of Wenzhou Medical University, Wenzhou, China; 3 Liver Cancer Institute, Zhongshan Hospital of Fudan University, Shanghai, China

**Keywords:** Hepatocellular carcinoma (HCC), Transarterial chemoembolization (TACE), Tumor Burden score (TBS), Prognostic model

## Abstract

**Background:**

Patients with hepatocellular carcinoma (HCC) who undergo transarterial chemoembolization (TACE) may have varied outcomes based on their liver function and tumor burden diversity. This study aims to assess the prognostic significance of the tumor burden score (TBS) in these patients and develop a prognostic model for their overall survival.

**Methods:**

The study involved a retrospective analysis of 644 newly diagnosed HCC patients undergoing TACE treatment. The individuals were assigned randomly to a training cohort (*n* = 452) and a validation cohort (*n* = 192). We utilized a multivariate Cox proportional risk model to identify independent preoperative predictive factors. We then evaluated model performance using the area under the curve (AUC), consistency index (c-index), calibration curve, and decision curve analysis (DCA) methods.

**Results:**

The multivariate analysis revealed four prognostic factors associated with overall survival: Tumor Burden Score, Tumor Extent, Types of portal vein invasion (PVI), and Child-Pugh score. The total score was calculated based on these factors. The model demonstrated strong discriminative ability with high AUC values and c-index, providing high net clinical benefits for patients. Based on the model’s scoring results, patients were categorized into high, medium, and low-risk groups. These results were validated in the validation cohort.

**Conclusions:**

The tumor burden score shows promise as a viable alternative prognostic indicator for assessing tumor burden in cases of HCC. The new prognostic model can place patients in one of three groups, which will estimate their individual outcomes. For high-risk patients, it is suggested to consider alternative treatment options or provide the best supportive care, as they may not benefit significantly from TACE treatment.

**Supplementary Information:**

The online version contains supplementary material available at 10.1186/s12885-024-12049-4.

## Background

Hepatocellular carcinoma (HCC) is the sixth most common cancer, and the second most lethal [1]. HCC ranks fourth in morbidity and second in mortality in China. It poses a serious threat to people’s health, with its high malignancy, rapid progression, poor prognosis, and a 5-year survival rate of only 12.1% [2]. Despite the improvements in screening and treatment over the years, a considerable proportion of patients are ineligible for surgical treatment at the time of diagnosis. According to many current HCC practice guidelines, transarterial chemoembolization (TACE) is considered one of the most commonly used local treatment modalities for intermediate-stage HCC [3, 4]. However, TACE is used in clinical practice beyond the guideline recommendations, not only for patients suffering from unresectable early HCC but also for those with liver-confined advanced disease [5–7]. Due to differences in tumor burden and liver reserve function among cancer patients, there may be heterogeneity in patients receiving TACE, which may result in variable outcomes [5, 8–10].

Researchers have explored the influencing factors of overall survival (OS) in HCC patients receiving TACE treatment, mainly including tumor characteristics and liver function [11]. Based on factors such as tumor size, number, liver function, and alpha-fetoprotein (AFP) level, researchers have developed several prognostic models, including the 6&12 score [5], HAP score [12], mHAP score [13], mHAPII score [14], mHAPIII score [15], and the pre-TACE prediction model [16]. However, these previous prognostic models have been questioned due to complex formulas, poor external validation results, and non-TACE populations [17–19], limiting their clinical application.

Recently, a new indicator called the “Tumor Burden Score (TBS)” has been proposed to stratify the risk of multifocal tumors [20]. The indicator considers tumor size and the number of tumors and has shown potential in predicting the prognosis of patients with colorectal liver metastasis, HCC, and intrahepatic cholangiocarcinoma who undergo surgical resection [20–23]. In this study, we created and validated a new model using preoperative TACE data in HCC patients. The model uses independent risk factors obtained from multivariate Cox analysis to determine the appropriateness of TACE as initial treatment.

## Methods

### Study population

**Fig. 1:**
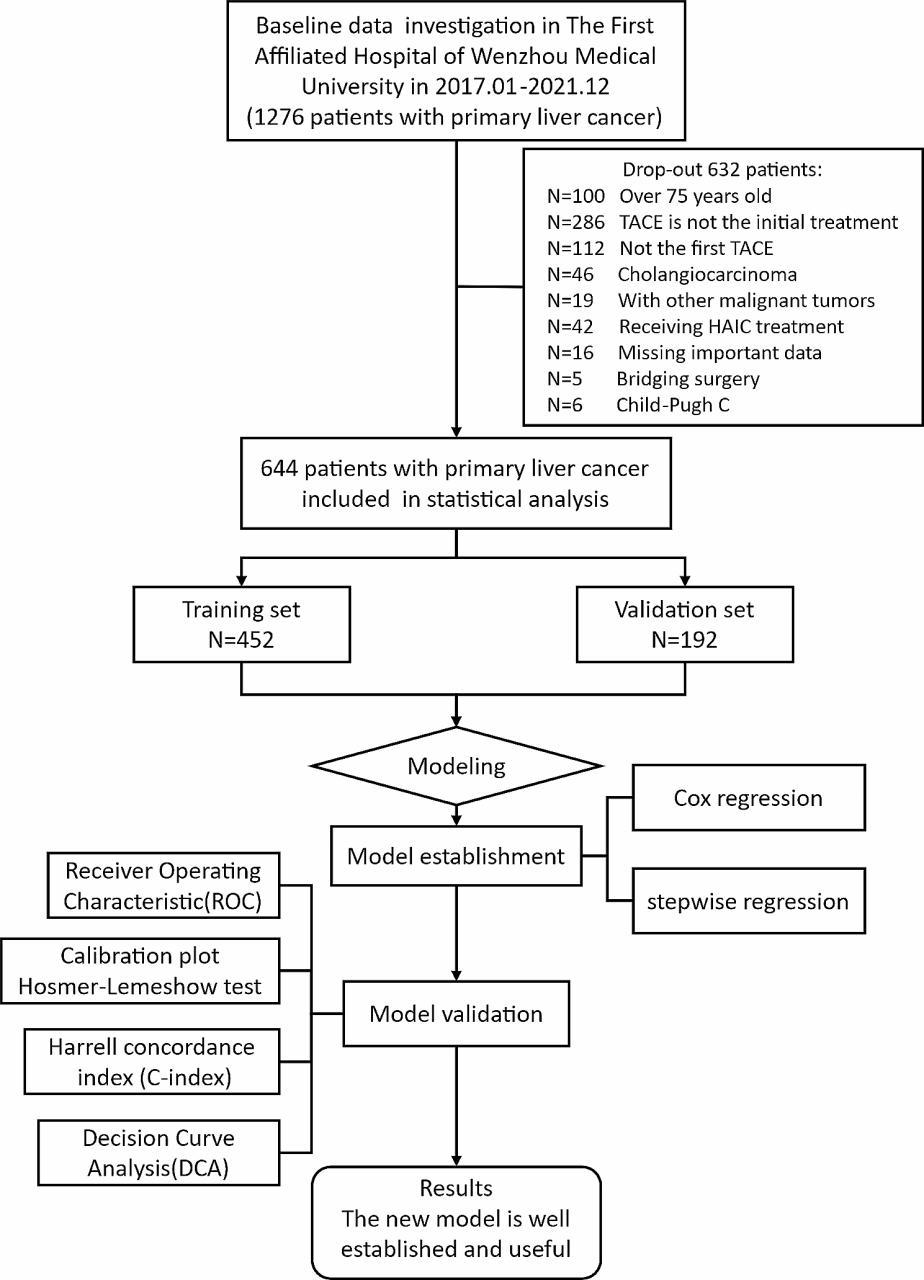
Diagram for inclusion of patients into the study. Abbreviations: TACE, transarterial chemoembolization; HAIC, hepatic artery infusion chemotherapy

A total of 1276 patients with HCC who received the TACE procedure were enrolled from January 2017 to December 2021 at the First Affiliated Hospital of Wenzhou Medical University. Eventually, 644 HCC patients were included according to the inclusion and exclusion criteria (Fig. 1). Based on a training cohort verification cohort ratio of approximately 7: 3, 452 patients were included in the training cohort and 192 patients in the validation cohort. The study protocol was approved by the Institutional Ethics Committee of the First Affiliated Hospital of Wenzhou Medical University. This study adhered to the ethical guidelines of the 1975 Declaration of Helsinki and was approved by the Ethics Committee of the First Affiliated Hospital of Wenzhou Medical University.

### Data collection

Clinical and sociodemographic data were collected from patients’ electronic medical records. Data captured included gender, age, etiology of hepatopathy (hepatitis B virus (HBV) infection or other causes), Barcelona Clinic Liver Cancer (BCLC) stage, China liver cancer staging (CNLC) stage, Child-Pugh score, presence of liver cirrhosis, presence of ascites, presence of extrahepatic metastasis (especially recorded lung metastasis and bone metastasis), level of AFP, and tumor characteristics such as maximum tumor size, number of liver lesions, lesion extent (unilobar or mutilobar), completeness of tumor capsule and types of portal vein invasion. The diagnosis of cirrhosis and PVI was based on clinical, radiological [computed tomography (CT) or magnetic resonance imaging (MRI)], and histological criteria.

**Fig. 2:**
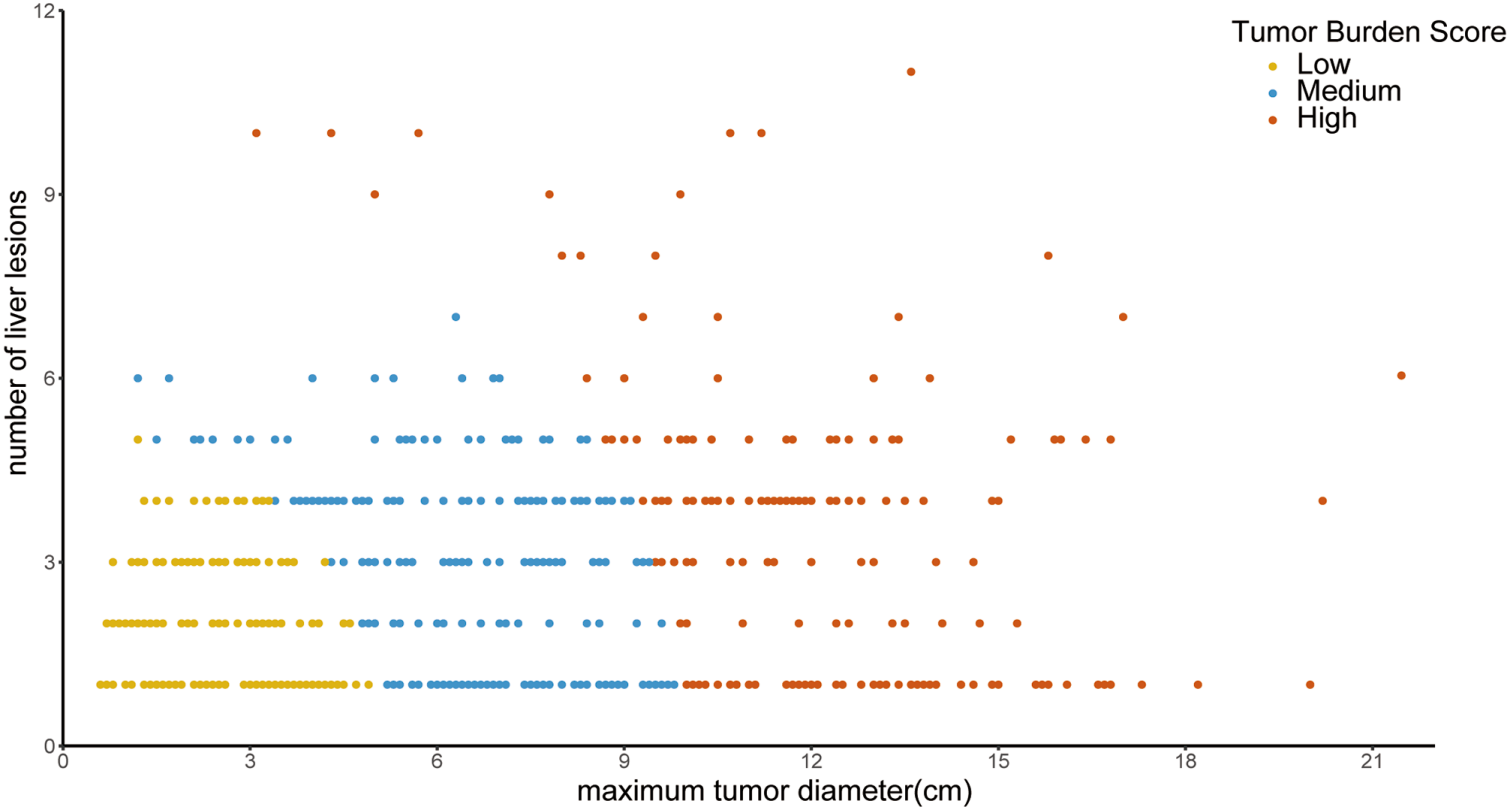
Scatter plot about maximum tumor diameter (x-axis) and number of intrahepatic tumors (y-axis), ${\left(\text{m}\text{a}\text{x}\text{i}\text{m}\text{u}\text{m} \text{t}\text{u}\text{m}\text{o}\text{r} \text{d}\text{i}\text{a}\text{m}\text{e}\text{t}\text{e}\text{r}\right)}^{2}+{\left(\text{n}\text{u}\text{m}\text{b}\text{e}\text{r} \text{o}\text{f} \text{l}\text{i}\text{v}\text{e}\text{r} \text{l}\text{e}\text{s}\text{i}\text{o}\text{n}\text{s}\right) }^{2}={\text{T}\text{B}\text{S}}^{2}$. Abbreviations: TBS, Tumor Burden Score

TBS was calculated using a combination of tumor size and the total number of tumors for each patient [20]. TBS was defined as the distance from the origin to a point on a plane, incorporating the maximum tumor diameter and the number of intrahepatic tumors. The formula used to calculate TBS was: (maximum tumor diameter)^2^ + (number of liver lesions)^2^=TBS^2^ (Fig. 2). Cut-off values for TBS were determined using X-tile, a retrospective bioinformatics tool developed by Camp and colleagues [24]. Patients were divided into three groups according to the TBS: high (over 10.00; 185, 28.6%), medium (5.20–10.00; 227, 35.4%), and low (less than 5.20; 269, 36.0%). Increasing TBS was associated with a progressively higher risk of death (referent low TBS, medium TBS, HR 3.20, 95% CI 2.39–4.28, *P* < 0.001; high TBS, HR 6.62, 95% CI 4.72–9.27, *P* < 0.001).

The primary outcome measure was OS, defined as the time from the date of TACE to the date of death or last follow-up. Patients who were alive at last follow-up or lost to follow-up were censored. Several scoring systems, namely 6&12 score [5], HAP score [12], mHAP score [13], mHAPII score [14], mHAPIII score [15], and the pre-TACE-Predict Model [16], were calculated based on their criteria.

### Treatment procedure

The experienced interventional radiologists performed the conventional TACE (cTACE) procedure. Firstly, they used the modified Seldinger technique to insert an angiographic catheter through the right femoral artery and access the hepatic artery. They then performed angiography of the common hepatic artery and superior mesenteric artery to visualize hepatic artery anatomy and portal vein patency. Next, a microcatheter was super-selected to the tumor-feeding artery, and then a mixture of chemotherapeutics and lipiodol was injected, the dosage of which was adjusted according to the number, maximum diameter, and embolization extent of the tumor lesions. Gelatin sponge or embolic microsphere was used for embolization until the tumor arterial flow was reduced on angiography. The chemotherapy drugs mainly include pirarubicin (10-50 mg), epirubicin (10-50 mg), mitomycin (10 mg), oxaliplatin (50-150 mg), raltitrexed (1-4 mg) and 5-fluorouracil (250-1000 mg). The range of lipiodol is 1 to 30 ml.

During the drug-eluting beads-TACE (DEB-TACE) procedure, the recommended injection rate for drug-loaded microspheres is 1 ml/min. The microspheres should be well distributed and evenly suspended during the injection process. When the flow rate of microspheres loaded with drug and contrast agent suspension in the artery has not emptied within 3–4 cardiac cycles, the injection should be stopped as it marked the endpoint of embolization. After a pause of 5 to 15 min, angiography was repeated. If tumor staining was still present, continue embolization until the endpoint of embolization was reached (tumor staining disappeared). Lesions that had not yet reached the endpoint of embolization could be further embolized with drug-loaded microspheres or blank microspheres, with a single embolization dose not exceeding 4 ml. Other particle embolic agents could be injected or re-embolized if economic conditions were limited.

TACE procedures would be repeated if viable tumor lesions or new emerging lesions were found by imaging examination in patients with tolerable physical conditions.

### Statistical analysis and model development

The presentation of quantitative variables following a normal distribution was expressed as mean ± standard deviation. Otherwise, they were described as the median and interquartile range (IQR). These data were compared using either Student’s t-test or non-parametric Mann-Whitney U test. Categorical variables were presented as their n (%) and compared using the Chi-squared test or Fisher’s exact test. In the training cohort, univariate Cox proportional hazards regression analysis was performed to determine the association of each parameter with all-cause mortality. A correlation heat map was performed to check the variables’ collinearity included in the previous step. A correlation coefficient greater than 0.7 indicated multicollinearity. Next, the variables with a *P* value less than 0.10 in the univariate regression analysis were included. Cox proportional hazards regression analysis with backward stepwise variable selection was used to extract independent predictors, while variables with collinearity were not included in the same model simultaneously. The new model was built by backward stepwise variable selection, with entry criteria set at the *P* < 0.05 level. To compare the performance of different models, the area under the curve (AUC) values were calculated, and the model with the highest AUC value was considered the final model. The β regression coefficients were multiplied by six and rounded to the nearest unit to obtain the score of the variable. The sum of the scores for each variable constituted the final score for each patient. Both TBS stratification and patient mortality risk stratification were carried out using x-tile software, a retrospective analysis tool. Statistical analysis was performed using R software (version 4.3.1, www.r-project.org). A two-sided *P* value less than 0.05 was considered statistically significant. The R packages used in this study are listed in Supplementary Table 1.

### Model evaluation and validation

To determine the accuracy of the predictive model, we used two methods: the area under the time-dependent receiver operating characteristic (ROC) curve and the concordance index (c-index). The ROC curve measures the predictive ability of the model over time, while the c-index estimates the probability that the predicted results match the actual results. Calibration, on the other hand, refers to the agreement between estimated and actual risk. We depicted the calibration curves to evaluate calibration. Another important consideration in clinical practice is the issue of benefits. We analyzed the benefits of the model using decision curve analysis (DCA) by comparing it to default strategies of treating all or no patients.

We evaluated the model using the above methods on both the training cohort and validation cohorts, comparing them with other models, like 6&12 score [5], HAP score [12], mHAP score [13], mHAPII score [14], mHAPIII score [15], and pre-TACE-Predict Model [16].

## Results

### Characteristics of training and validation cohorts

**Fig. 3:**
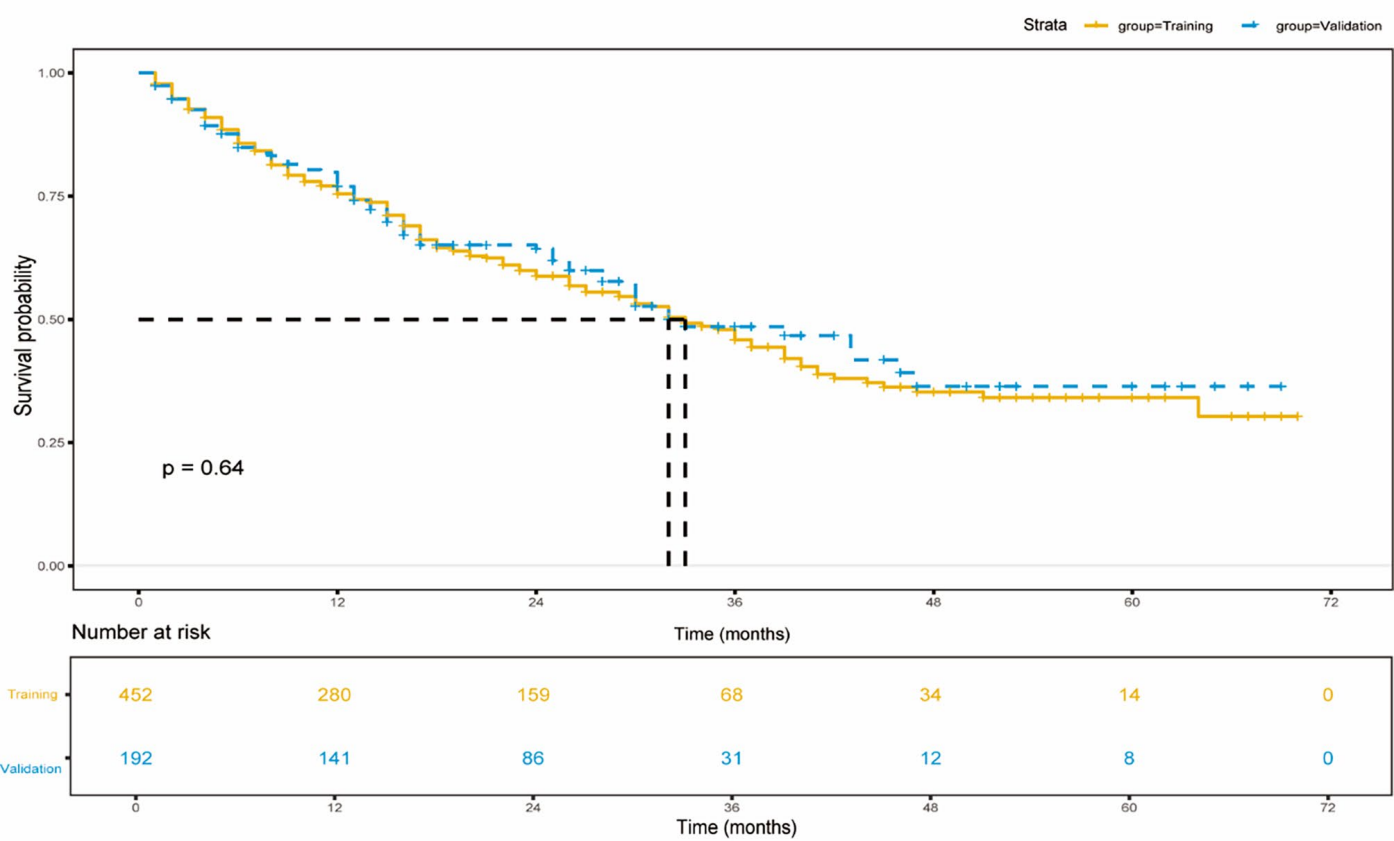
Kaplan-Meier survival curves of the training cohort and validation cohort

A total of 644 patients met the eligibility criteria and were included in the final cohort and randomized into training (N1 = 452, 70%) and validation (N2 = 192, 30%) cohorts (Fig. 1). The baseline characteristics of the two cohorts were comparable, and there was no significant difference between patients (Table 1). 85% of the enrolled patients were male, 15% were female, and the median age was 59.5 years old. HBV was the main cause of the patients, accounting for 79%, and 75% of patients had liver cirrhosis. The liver function grading was mainly distributed in A5, A6, and B7. In terms of tumor characteristics, the median of the maximum diameter of HCC was 6.1 cm (IQR: 3, 9.7), and most patients did not have portal vein invasion or extrahepatic metastasis. TBS was almost evenly distributed among the low, medium, and high groups. In a subgroup analysis based on the BCLC stage, significant differences in OS were observed among high, medium, and low TBS patients in the BCLC stage B/C group. Patients with high TBS had the worst prognosis in the BCLC stage B/C group (Supplementary Fig. 1B). However, it is important to note that TBS did not significantly affect BCLC stage 0/A stage (Supplementary Fig. 1A). The median OS for the training cohort was 33 months (95% CI: 29, 39), and 32 months (95% CI: 30, NA) for the internal validation cohort (Fig. 3).

**Table 1:** Characteristics of patients in the training and validation cohort

Variables	Total (*n* = 644)	Training group (*n* = 452)	Validation Group (*n* = 192)	*P* value
Gender				0.808
Male	545 (85)	381 (84)	164 (85)	
Female	99 (15)	71 (16)	28 (15)	
Age (years)	59.5 (53, 68)	59 (51, 67)	60 (54, 68)	0.173
Etiology				0.145
HBV	511 (79)	366 (81)	145 (76)	
Others	133 (21)	86 (19)	47 (24)	
BCLC-stage				0.115
0	47 (7)	31 (7)	16 (8)	
A	199 (31)	128 (28)	71 (37)	
B	211 (33)	156 (35)	55 (29)	
C	187 (29)	137 (30)	50 (26)	
CNLC-stage				0.181
Ia	112 (17)	73 (16)	39 (20)	
Ib	140 (22)	90 (20)	50 (26)	
IIa	69 (11)	51 (11)	18 (9)	
IIb	136 (21)	101 (22)	35 (18)	
IIIa	145 (23)	103 (23)	42 (22)	
IIIb	42 (7)	34 (8)	8 (4)	
Ascite				0.982
No	461 (72)	324 (72)	137 (71)	
Mild	165 (26)	115 (26)	50 (26)	
Moderate and above	16 (2)	11 (2)	5 (3)	
Liver cirrhosis				0.956
No	160 (25)	113 (25)	47 (24)	
Yes	483 (75)	338 (75)	145 (76)	
AFP				0.28
≤ 400	390 (64)	267 (63)	123 (68)	
>400	215 (36)	157 (37)	58 (32)	
Child-Pugh score				0.725
5	270 (42)	189 (42)	81 (43)	
6	183 (29)	135 (30)	48 (25)	
7	121 (19)	83 (19)	38 (20)	
8	46 (7)	30 (7)	16 (8)	
9	17 (3)	11 (2)	6 (3)	
Tumor size(cm)	6.1 (3, 9.7)	6.1 (3.1, 9.8)	6.1 (3, 9.43)	0.687
Tumor number	2 (1, 4)	3 (1, 4)	2 (1, 4)	0.058
Tumor Burden Score				0.784
Low	232 (36)	159 (35)	73 (38)	
Medium	227 (35)	162 (36)	65 (34)	
High	185 (29)	131 (29)	54 (28)	
Tumor extent				0.053
Unilobar	425 (67)	288 (65)	137 (73)	
Mutilobar	209 (33)	158 (35)	51 (27)	
Tumor capsule				0.236
Complete	298 (46)	202 (45)	96 (50)	
Incomplete	344 (54)	249 (55)	95 (50)	
Types of PVI				0.698
No	496 (77)	344 (76)	152 (79)	
I(branch)	97 (15)	71 (16)	26 (14)	
II(trunk)	51 (8)	37 (8)	14 (7)	
Extrahepatic metastasis				0.161
No	602 (93)	418 (92)	184 (96)	
Yes	42 (7)	34 (8)	8 (4)	
Lung meta				0.25
No	630 (98)	440 (97)	190 (99)	
Yes	14 (2)	12 (3)	2 (1)	
Bone meta				1
No	637 (99)	447 (99)	190 (99)	
Yes	7 (1)	5 (1)	2 (1)	
Number of TACE				1
1	231 (36)	162 (36)	69 (36)	
≥ 2	413 (64)	290 (64)	123 (64)	
Types of TACE				0.612
conventional TACE	555 (86)	387 (86)	168 (88)	
drug-eluting beads TACE	89 (14)	65 (14)	24 (12)	

### Building a prognostic model in training cohort

**Fig. 4:**
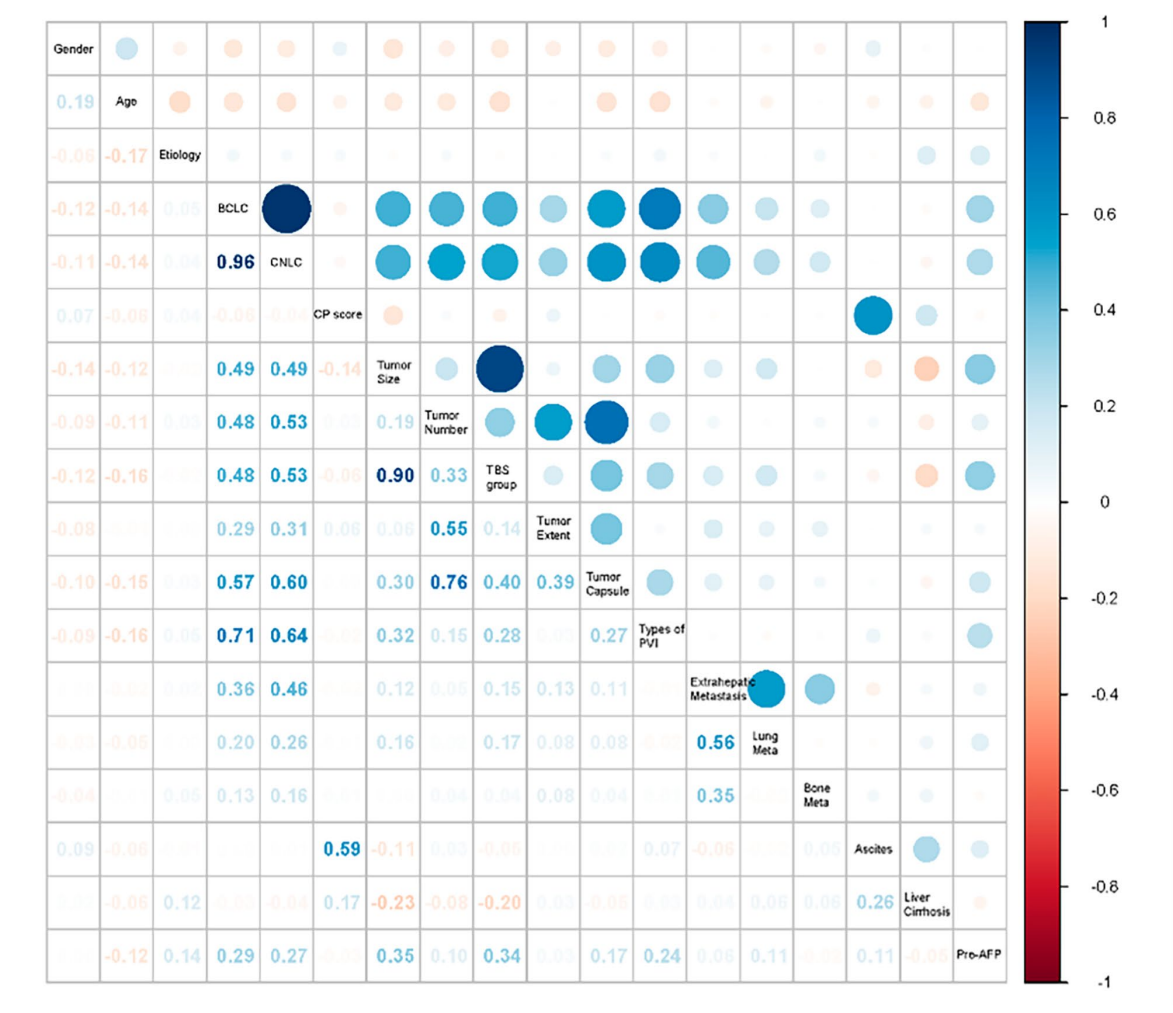
Correlation heat map between various factors

Before constructing our model, we conducted univariate and multivariate analyses of prognostic factors in our inclusive population. Based on the results of univariate analysis (Table 2), BCLC-stage, CNLC-stage, Child-Pugh score, tumor size, tumor number, tumor burden score, tumor extent, tumor capsule, types of PVI, bone meta, ascites, and AFP were correlated with OS (*p* < 0.05). Subsequently, we completed the correlation analysis. We defined a correlation coefficient greater than 0.7 as a strong correlation. We found a strong correlation between BCLC-stage and CNLC-stage, tumor size and TBS, tumor number and tumor capsule, and BCLC-stage and types of PVI (Fig. 4). For factors with strong correlation, we selected only one from each group for permutation and combination with other factors. Then we performed multiple stepwise regression analyses and ultimately selected the model with the highest AUC value obtained as our final model (Table 2).

After a backward stepwise removal of variables, four remained significant for OS: Child-Pugh score, TBS, Tumor extent, and Types of PVI (*p* < 0.05). We constructed the model using two methods integrating these predictors. One used the nomogram to predict the 1-, 2-, 3-, and 5-year OS (Supplementary Fig. 2), and the other used the calculated β values (regression coefficient) of variables derived from stepwise Cox regression analysis multiplied by 6 and rounded to get a new score (Table 3). Comparing the AUC values of the two models, it was found that the second assignment scoring model had a higher AUC value (Supplementary Figs. 4 and 10A). Therefore, we selected the second model as our final model.

**Table 2:** Univariate and multivariate Cox proportional hazard analysis of OS in the training cohort

Variables	Univariate analysis				Multivariate analysis		
	Hazard Ratio	95%CI	*p* value		Hazard Ratio	95%CI	*p* value
Gender (Male vs. Female)	0.98	0.66–1.45	0.926				
Age (years)	1	0.99–1.02	0.917				
Etiology (Others vs. HBV)	1.15	0.8–1.65	0.464				
BCLC-stage							
0	Ref						
A	3.47	1.25–9.63	0.017				
B	6.07	2.21–16.63	< 0.001				
C	11.31	4.1-31.18	< 0.001				
CNLC-stage							
Ia	Ref						
Ib	2.08	1.15–3.77	0.015				
IIa	2.14	1.09–4.21	0.027				
IIb	4.18	2.38–7.33	< 0.001				
IIIa	6.5	3.7-11.42	< 0.001				
IIIb	4.91	2.34–10.32	< 0.001				
Child-Pugh score							
5	Ref				Ref		
6	1.32	0.94–1.87	0.111		1.25	0.87–1.79	0.222
7	1.55	1.05–2.28	0.027		1.99	1.34–2.96	0.001
8	2.07	1.18–3.62	0.011		2.12	1.2–3.76	0.01
9	1.89	0.76–4.71	0.169		3.34	1.32–8.45	0.011
Tumor size	1.13	1.09–1.16	< 0.001				
Tumor Number	1.27	1.19–1.37	< 0.001				
Tumor Burden Score							
Low	Ref				Ref		
Medium	2.66	1.83–3.86	< 0.001		2.42	1.62–3.62	< 0.001
High	5.78	3.9–8.57	< 0.001		5.38	3.44–8.41	< 0.001
Tumor extent (Mutilobar vs. Unilobar)	1.65	1.24–2.21	0.001		1.46	1.07–2.01	0.019
Tumor capsule (Yes vs. No)	2.37	1.76–3.2	< 0.001		1.22	0.86–1.73	0.264
Types of PVI							
No	Ref				Ref		
I(branch)	2.77	1.93–3.96	< 0.001		1.93	1.3–2.86	0.001
II(trunk)	2.81	1.71–4.63	< 0.001		2.82	1.67–4.74	< 0.001
Extrahepatic metastasis (Yes vs. No)	1.61	0.91–2.85	0.101				
Lung meta (Yes vs. No)	1.96	0.72–5.32	0.185				
Bone meta (Yes vs. No)	4.57	1.44–14.53	0.01				
Ascites							
No	Ref						
Mild	1.64	1.21–2.23	0.002				
Moderate and above	1.17	0.37–3.67	0.793				
Liver cirrhosis (Yes vs. No)	0.76	0.55–1.04	0.085				
AFP (ng/ml) (> 400 vs. ≤ 400)	1.77	1.32–2.38	< 0.001				
Number of TACE(≥2 vs. 1)	0.58	0.43–0.77	< 0.001				
Types of TACE							
(DEB-TACE vs. c-TACE)	1.26	0.84–1.88	0.26				

**Table 3:** β-coefficient and corresponding rounded risk score from multivariate Cox regression model in the training cohort

Variables		Overall survival			point	*p* value
		HR	95%CI	β-coefficient		
Child-Pugh score	5,6	Ref				
	7	1.99	1.34–2.96	0.69	4	0.001
	8	2.12	1.2–3.76	0.74	4	0.01
	9	3.34	1.32–8.45	1.21	7	0.011
Tumor Burden Score	Low	Ref				
	Medium	2.42	1.62–3.62	0.89	5	< 0.001
	High	5.38	3.44–8.41	1.68	10	< 0.001
Tumor extent	unilobar	Ref				
	Mutilobar	1.46	1.07–2.01	0.38	2	0.019
Types of PVI	No	Ref				
	I(branch)	1.93	1.3–2.86	0.66	4	0.001
	II(trunk)	2.82	1.67–4.74	1.04	6	< 0.001

### Performance assessment and validation

**Fig. 5:**
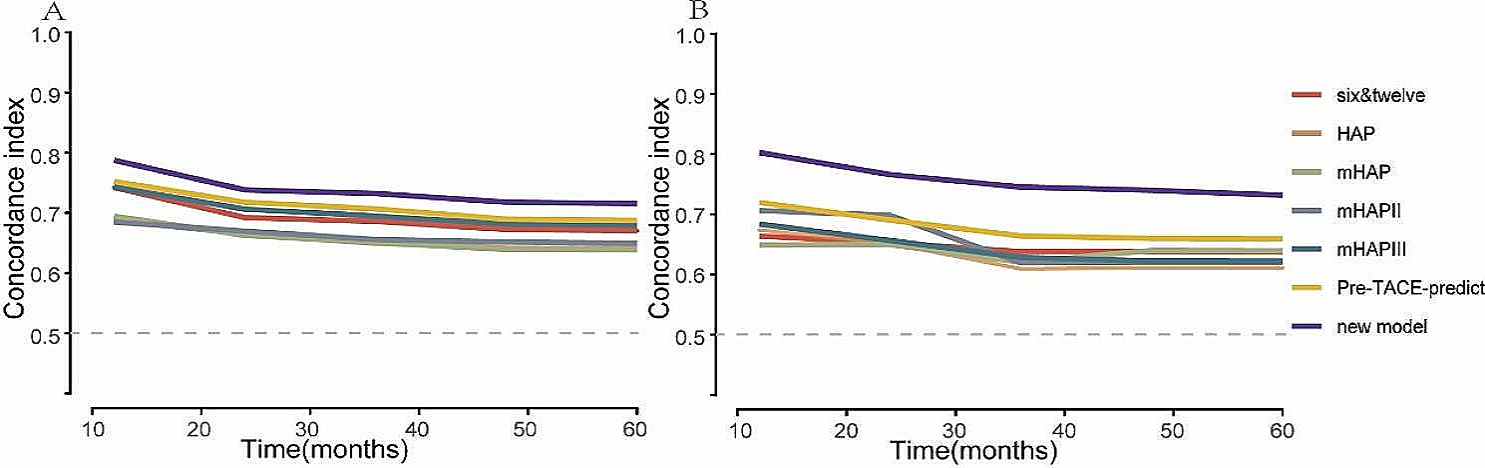
Time-dependent concordance index curve of the new model in the training and validation cohort. (**A**) The c-index for the training cohort. (**B**) The c-index for the validation cohort

**Fig. 6:**
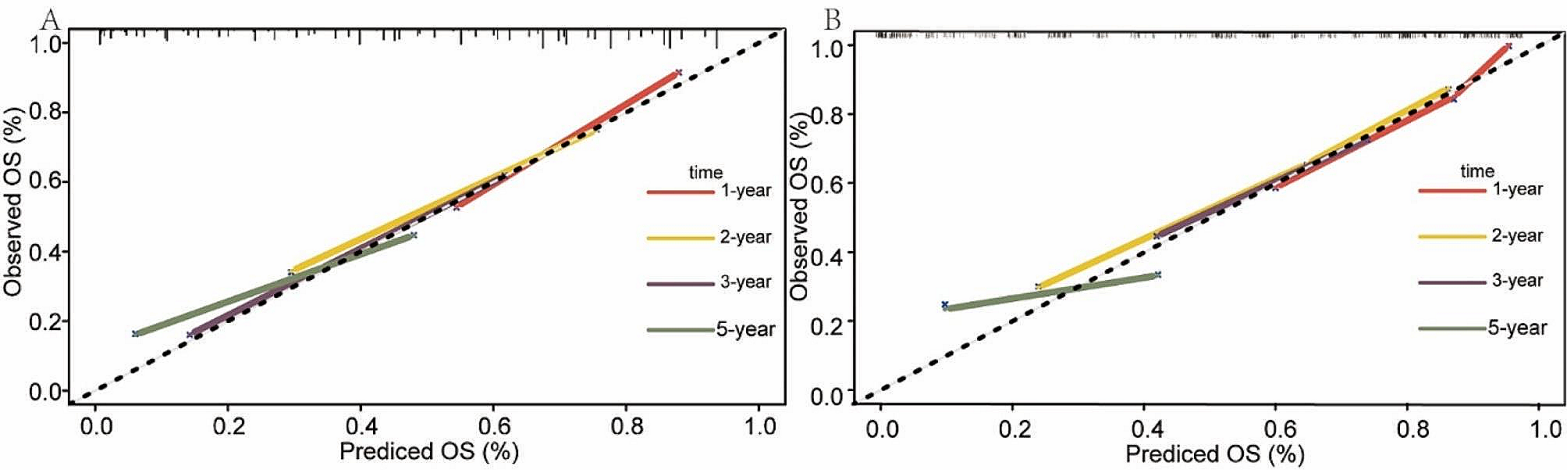
The plots of calibration of 1-year, 2-year, 3-year, and 5-year overall survival. Calibration curves of my model in training cohort (**A**). Calibration curves of my model in the validation cohort (**B**). The dotted lines represent the ideal predictive model, and the solid red line represents the observed model

**Fig. 7:**
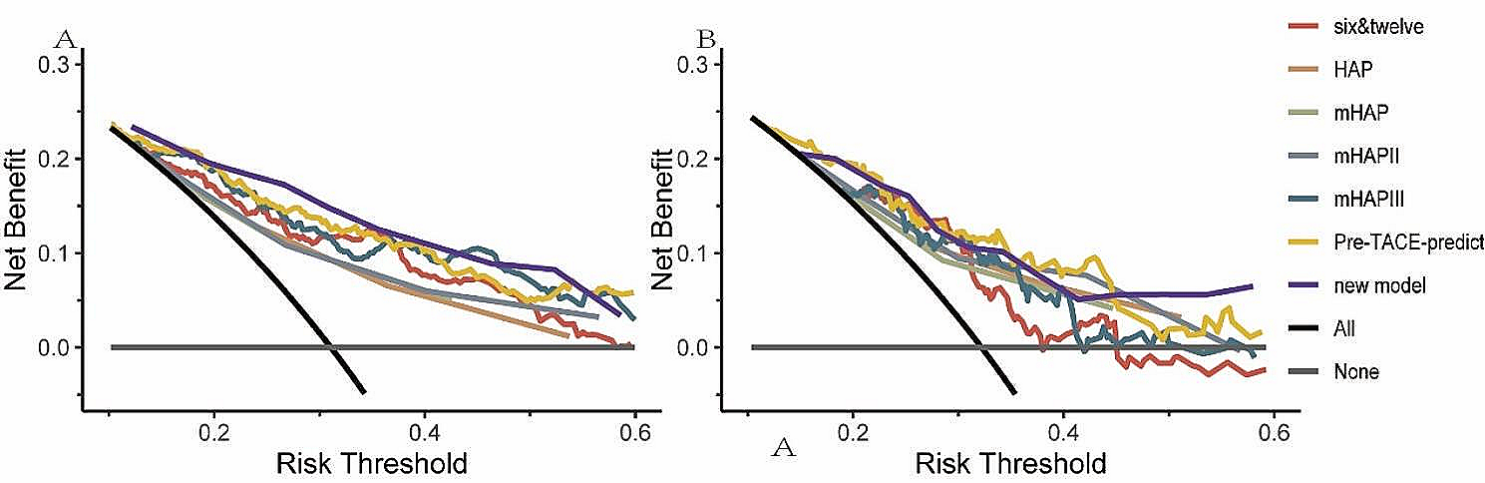
Clinical decision curve analysis for the training cohort (**A**) and validation cohort (**B**) for clinical benefits. The black line represents the net benefit of the strategy of treating all patients. The gray line represents the net benefit of the strategy of treating no patients

ROC analysis and c-index are commonly used as a stick to evaluate the predictive performance of clinical research, namely discrimination [25, 26]. The AUCs for 1-, 2-, 3-, and 5-year OS in the training cohort were 0.847, 0.803, 0.777, and 0.768, respectively (Fig. 10A and C). Similarly, in the validation cohort, the AUCs for 1-, 2-, 3-, and 5-year OS were 0.862, 0.845, 0.788, and 0.733, respectively (Fig. 10B and D). The C-indexes for OS prediction in the training and validation cohorts were 0.75 and 0.75 respectively. The curve of the c-index of the training and validation cohorts over time is shown in Fig. 5. The values for 1-, 2-, 3-, and 5-year were 0.787, 0.738, 0.732, and 0.715 for the training cohort, while the validation cohort were 0.803, 0.766, 0.745 and 0.732, respectively. Another evaluation method is calibration, which compares the predicted survival rate to the actual survival rate. The calibration curve visually demonstrates the accuracy of the model’s predictions [27]. Figure 6 showed that the predicted OS results of the 1-, 2-, 3-, and 5-year survival have good consistency with actual observations both in the training and validation cohorts. In addition to measures such as discrimination and calibration, DCA is an important verification tool for evaluating the clinical benefits of a model and comparing multiple models to determine the best decision-making model [28]. Figure 7 shows that our model has a significant net clinical benefit in predicting survival.

**Fig. 8:**
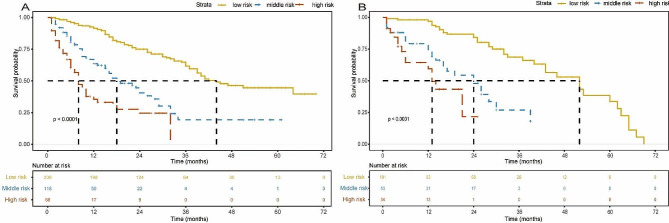
Kaplan-Meier survival curves of patients with HCC stratified by the new model in the training cohort (**A**) and in the validation cohort (**B**)

**Fig. 9:**
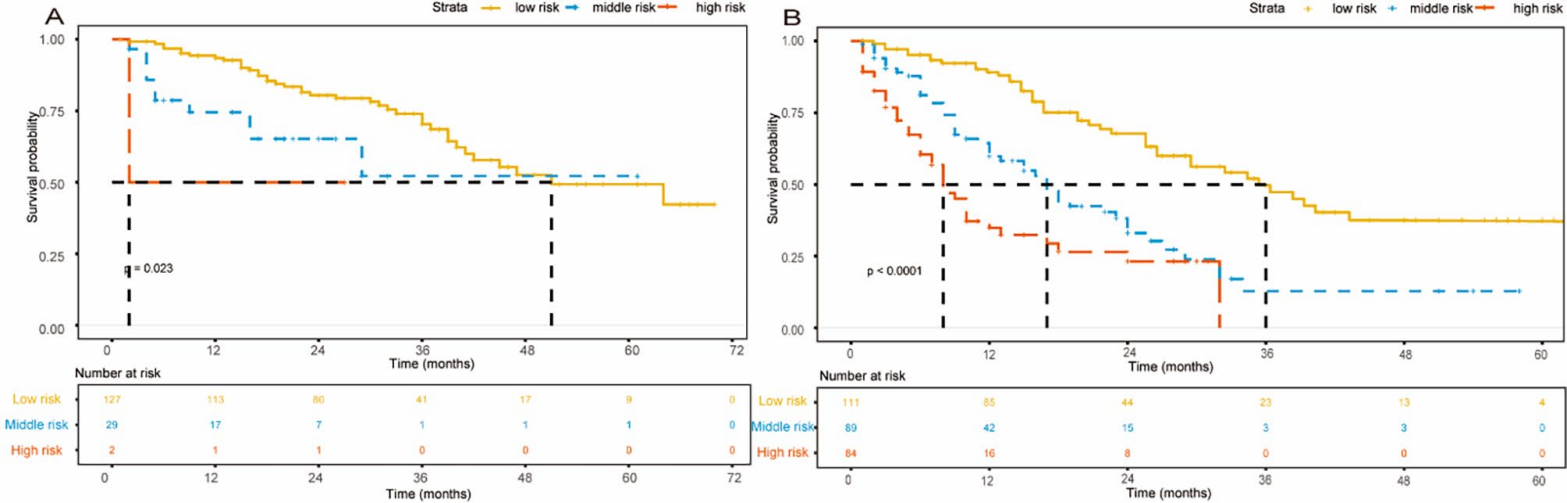
Kaplan-Meier survival curves of patients in the training cohort stratified by the new model in the BCLC 0/A (**A**) and BCLC B/C (**B**)

HCC patients were classified into three levels using x-tile based on the total score obtained: low-risk group (0–7), medium-risk group (8–12), and high-risk group (greater than 13). Kaplan-Meier curves of the two cohorts are shown in Fig. 8. For the low, the medium OS was 44 months (95% CI, 39-NA); for the medium and the high, the medium OS was 18 months (95% CI, 16–27) and 8 months (95% CI, 6–13), respectively (*p* < 0.0001). In the validation cohort, the median OS was 52 months (95% CI, 43–63) for the low-risk group, 24 months (95% CI, 16-NA) for the medium-risk group, and 13 months (95% CI, 6-NA) for the high-risk group (*p* < 0.0001). In the training cohort, there was a significant statistical difference in OS among three different risk stratification groups for BCLC stage 0/A patients (log-rank *p* < 0.0001, Fig. 9A), and for BCLC stage B/C (log-rank *p* < 0.0001, Fig. 9B). The same results were obtained in the validation group (Supplementary Fig. 3).

### Comparison of different scoring systems

**Fig. 10:**
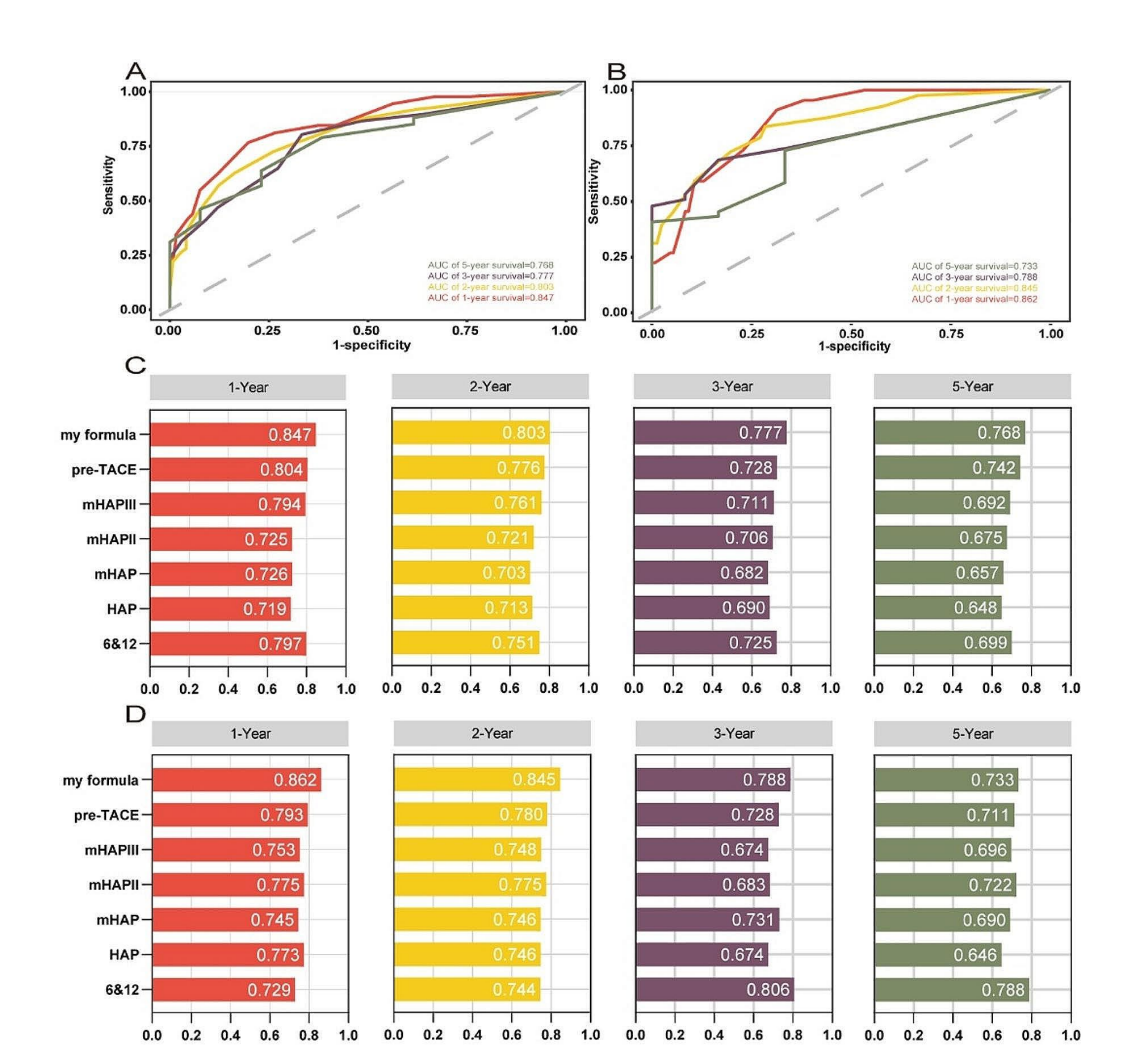
Time-dependent ROC curve for predicting OS at 1-, 2-, 3-, and 5-year in the training cohort (**A**) and in the validation cohort (**B**). Comparison of time-dependent AUC values of different prediction models at 1-, 2-, 3-, and 5-year in the training cohort (**C**) and the validation cohort (**D**)

As previously mentioned, both AUC and c-index are used to evaluate discrimination ability. DCA provides a clear demonstration of the net clinical benefits of models for patients. We calculated the results of several other models, including 6&12 score [5], HAP score [12], mHAP score [13], mHAPII score [14], mHAPIII score [15], and pre-TACE-Predict Model [16]. Our model consistently had AUC values higher than 0.80 for both 1-year and 2-year in both cohorts, surpassing the AUC values of other models. In the training cohort, the AUC values of the 3-year and 5-year were higher than 0.75, outperforming other models. Similarly, in the validation cohort, our model had higher AUC values for 3-year and 5-year compared to most models (Fig. 10C and D).

The C-indexes of these models were also calculated. The HAP, mHAP, and mHAPII models had C-index values below 0.7 in the training cohort, while our model consistently had C-index values above 0.7, surpassing other models (Fig. 5). Regarding the validation cohort, the C-index values of 6&12, HAP, mHAP, and mHAPIII were all below 0.7. Our prediction model was above 0.7 and performed better than other models.

Our predictive model showed a better ability to predict survival compared to the all-patients dead scheme and the no-patients dead scheme, as shown by the DCA curve in Fig. 7. The DCA curve demonstrated that our model yielded greater net benefit improvement compared to other prognostic evaluation systems.

## Discussion

The high malignancy, rapid progression, and poor prognosis have seriously threatened the lives and health of our people. TACE is the most commonly used treatment method for mid-term HCC [3, 4]. However, TACE is also applied to patients suffering from unresectable early HCC and those with liver-confined advanced diseases [5–7]. However, due to differences in tumor burden and liver reserve function, patients often have different prognosis [5, 8–10]. This heterogeneity makes prognosis prediction challenging, therefore making treatment decisions for these patients remains difficult. Despite the development of numerous prognostic models to predict the effectiveness of TACE treatment, there is currently no standardized policy to determine which patients can benefit from TACE. The objective of this study is to construct and verify a new model for predicting the prognostic role of HCC patients receiving TACE.

When the Milan Criteria (MC) was first proposed in 1996, they quickly became the cornerstone for selecting and managing liver cancer patients. However, due to the variable tumor burden, significant differences in treatment choices may be present for patients who exceed MC. To address this, up-to-7 criteria and up-to-11 criteria for evaluating the burden of liver cancer have been proposed. These two models combine the maximum nodule diameter and the number of nodules, with a total of no more than 7 or 11. Unlike the traditional method of using the maximum diameter and the number of nodules as the burden of liver cancer, TBS has recently been proposed to minimize the heterogeneity of cancer patients. Duvoux’s team proposed a French AFP liver transplantation model consisting of maximum tumor diameter, number of tumors, and AFP level, with transplant eligibility of ≤ 2 points. As long as the AFP is ≤ 100 (0 points) and the maximum tumor diameter is ≤ 3 cm (0 points), the patient can be put on the waiting list regardless of the number of tumors in the patient [29], which means that patients can easily be on the waiting list, provided they have micronodular HCC and are AFP negative. However, a later study by Mazzotta found that there was a significant difference in 3-year and 5-year survival among those with AFP ≤ 2 grouped with a cutoff of 5 after entering the waiting list and before undergoing liver transplantation [30]. Tumor diameter and number were found to have important prognostic value in liver transplant patients. TBS is an index that combines them to reduce the bias in prognostic estimation due to the truncated values described above. TBS is a promising tool for predicting the outcomes of HCC patients after liver transplantation. A study by Moris D et al. showed that patients with high TBS had worse OS and recurrence free survival. When the study superimposed TBS on the Milan standard, it was found that patients with higher TBS values within the Milan standard had a higher risk of recurrence [23]. Further research is needed to determine whether our TBS-based model can be extended to patients with other treatment options. It has been proven to have excellent prognostic discrimination in colorectal liver metastasis, liver cancer resection, and liver transplant patients [20, 22, 23, 31].

In this study, we performed an OS analysis on the TBS values of all the patients included. The results showed that it could distinguish the OS of liver cancer patients receiving TACE, consistent with previous research results [21].

In subgroup analysis based on BCLC stage, it was found that patients with high TBS had the worst prognosis in the BCLC B/C. However, TBS did not play a role in the early BCLC stage. According to the definition of BCLC, BCLC 0/A refers to a single nodule or multiple small nodules. So even if the patient is in the high or medium TBS group, the increase in TBS is mostly caused by isolated large nodules, TACE treatment still has a nice effect on the tumor-feeding artery, leading to a good prognosis, similar to the conclusions obtained in previous studies [21].

We then added tumor extent (Mutilobar vs. Unilobar) and PVI level (no or below branches or above branches) to the TBS to jointly form tumor features. Combining with the Child-Pugh score, we constructed a prognostic prediction model that demonstrated excellent survival prediction performance. As the Child-Pugh score, TBS, tumor distribution increase, and the grade of PVI increases, the survival prognosis of patients will deteriorate. We validated the model in the training and validation cohorts using AUC and C-index, and the validation cohort had even better discriminative ability with higher AUC and C-index values. We also used a nomogram-like approach to estimate patient survival based on patient scores, which created a graphical statistical prognostic model that can predict the probability of death over time (Supplementary Fig. 5) [32, 33]. From this, we can see that as patient scores increase, the survival rate gradually deteriorates. In clinical practice, we can infer the survival rate of patients at different times in their basic clinical data. Moreover, we divided HCC patients into three different risk groups based on the predicted risk of death using the model. There was a significant difference in survival among the three groups in both the training and validation cohorts. Additionally, survival analysis on the training and validation cohorts showed that our model has prognostic stratification ability for both early and advanced HCC. With the continuous development of clinical trials of new drugs [34, 35], our model could guide the identification of patients who may not benefit from TACE treatment, allowing for timely shifts to systematic or evidence-based treatment when poor patient survival outcomes were estimated. Therefore, our prediction model may have sufficient external utility and potential for further clinical application in the future.

Our scoring system includes risk factors consistent with previous research. TBS represents the maximum diameter of the tumor and the number of intrahepatic tumors. To our knowledge, our study was the first to incorporate TBS into the TACE treatment prognosis model for HCC, providing solid evidence that TBS has good discrimination ability as a tumor burden indicator. PVI and tumor extension represent the invasiveness of tumor biology [36–39]. Unlike other prognostic models for solid tumors, liver reserve function plays an important role in disease analysis and prognosis of patients. The Child-Pugh score represents liver reserve status and evaluates liver tolerance to TACE, widely used in liver reserve function evaluation and predictive systems [4, 40, 41]. The traditional liver cancer monitoring indicator AFP did not have significant prognostic value in this study. It remained a controversial biomarker for liver cancer, as many patients exhibit negative AFP and its sensitivity and specificity are not high, similar to some previous research findings [42, 43]. Our analysis showed that our scoring system predicted the survival of TACE-treated patients better than other scoring systems. The model has the highest discriminatory ability to predict OS due to its maximum AUC value and c-index while providing relatively higher clinical net benefits for patients from the DCA results. Above all, our model has the following advantages. Firstly, it includes simple clinical indicators that are easily obtainable. Secondly, it can be applied to patients outside of TACE guidance standards. Lastly, the probability of specific survival time can be provided to clinical doctors.

As our model demonstrates prognostic stratification significance for HCC patients receiving TACE at different stages, it is important to note that for HCC patients treated with other methods, such as surgery, radiofrequency, liver transplantation, etc., they cannot be validated from our inclusion population due to limitations in inclusion and exclusion criteria. For patients undergoing surgical resection treatment, OS gradually deteriorates with increasing TBS. Regardless of the BCLC stage, there was no difference in OS between patients with similar TBS groups (BCLC-A/medium TBS and BCLC-B/medium TBS, *P* = 0.930; BCLC-A/high TBS and BCLC-B/high TBS, *P* = 0.175). Patients with BCLC-B HCC with medium TBS have better OS than those with BCLC-A with high TBS [22].

This study has certain limitations that should be considered when interpreting the results. Like all retrospective studies, there may be selection bias in determining which patients receive TACE treatment. Moreover, most of the included patients had HBV infection-related liver cancer, so we still need more external validation when extrapolating the results to other etiological populations. Additionally, the validation group used in our research is from the same institution, so external data validation from multiple centers is necessary. Lastly, this study only focused on patients receiving TACE as their initial treatment. Studying patients who have previously received other treatments, such as surgery or radiofrequency ablation, may broaden the application scope of the model.

## Conclusion

In summary, this study developed a simple, clinically relevant, and easily accessible model to predict the prognosis of patients receiving TACE treatment, which includes three tumor features and one liver function indicator. One of the indicators, TBS, is entering the TACE prognostic model for the first time. From the model prediction, high-risk patients may not benefit from TACE treatment and should consider alternative treatments. This model has been validated in the validation cohort and performs well compared to other prognostic systems, but it still needs to be prospectively validated in a larger cohort to confirm the suitability of our findings.

### Electronic supplementary material

Below is the link to the electronic supplementary material.

Supplementary Material 1

## Data Availability

The datasets used and/or analyzed during the current study are available from the corresponding author on reasonable request.

## Abbreviations

HCC: Hepatocellular carcinoma
TACE: Transarterial chemoembolization
TBS: Tumor burden score
AUC: Area under the curve
c-index: Consistency index
DCA: Decision curve analysis
PVI: Portal vein invasion
OS: Overall survival
AFP: Alpha-fetoprotein
HBV: Hepatitis B virus
BCLC: Barcelona Clinic Liver Cancer
CNLC: China liver cancer staging
CT: Computed tomography
MRI: Magnetic resonance imaging
cTACE: Conventional TACE
DEB-TACE: Drug-eluting beads TACE
IQR: Interquartile range
ROC: Receiver operating characteristic
