# Supplementary material for: Construction of a prognostic model for hepatocellular carcinoma patients receiving transarterial chemoembolization treatment based on the Tumor Burden Score
Source: BMC Cancer. 2024 Mar 6;24:306. doi: 10.1186/s12885-024-12049-4 (PMC10916036; doi:10.1186/s12885-024-12049-4)
Supplement: Supplementary file 1 — Supplementary Material 1 [file 12885_2024_12049_MOESM1_ESM.docx]

Supplementary Table 1 The R Packages Used for Statistical Analysis

| R package |  | statistical analysis |
| --- | --- | --- |
| timeROC |  | ROC curve analysis; compare AUC |
| pec |  | c-index |
| ggplot2 |  | Plot |
| rms |  | Nomogram construction and calibration |
| rmda |  | Decision curve analysis |

AUC = area under the curve, ROC = receiver operating characteristic, c-index= the concordance index.


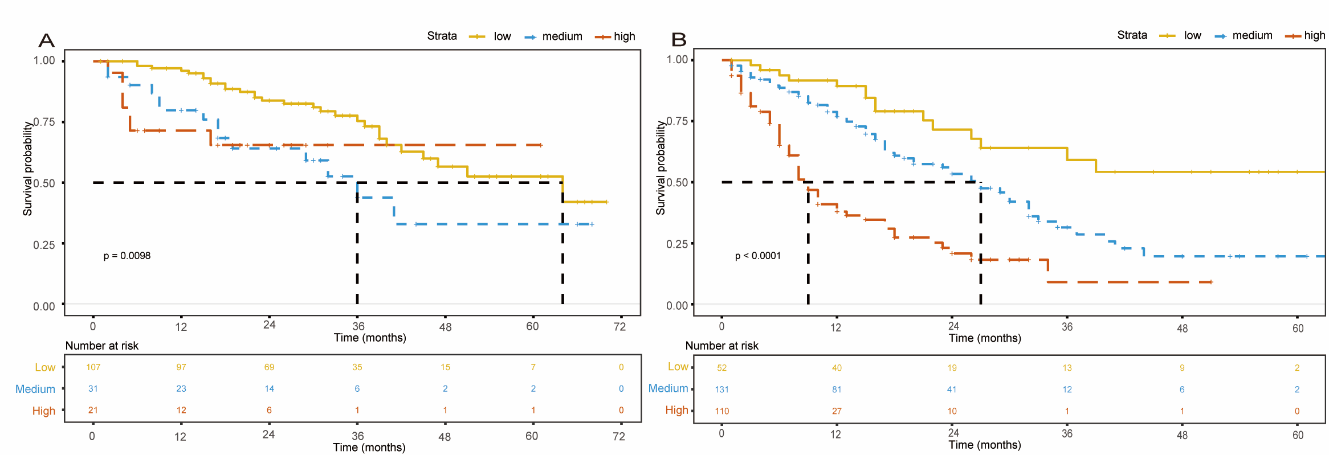


Supplementary figure 1 Kaplan-Meier survival curves of patients in the training cohort stratified by TBS in the BCLC 0/A (A) and BCLC B/C (B).





Supplementary figure 2 Nomogram to calculate risk score and predict survival probability


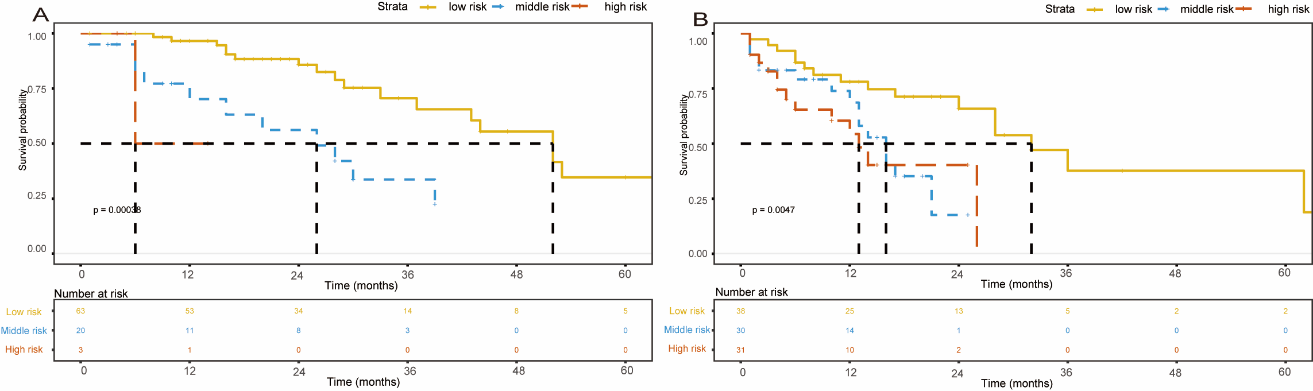


Supplementary figure 3 Kaplan-Meier survival curves of patients in the validation cohort stratified by the new model in the BCLC 0/A (A) and BCLC B/C (B).
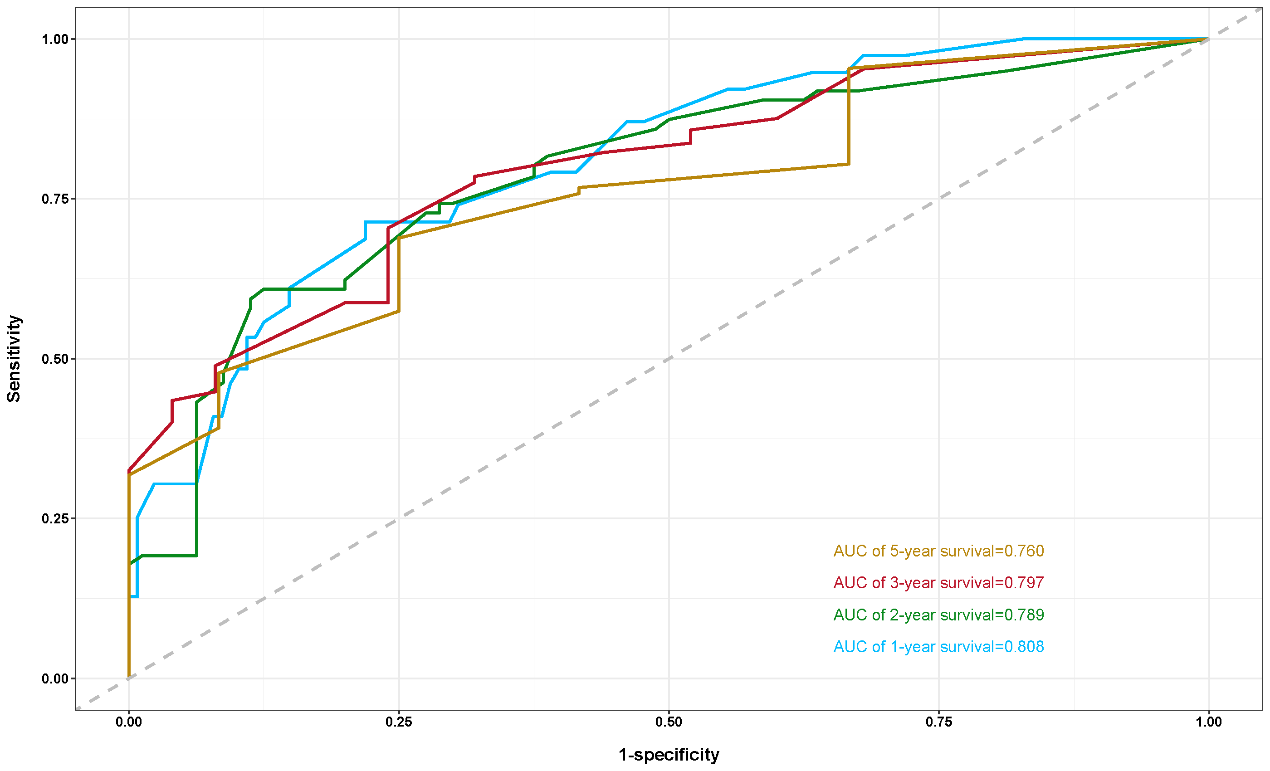


Supplementary figure 4 ROC curves of the nomogram prediction model.


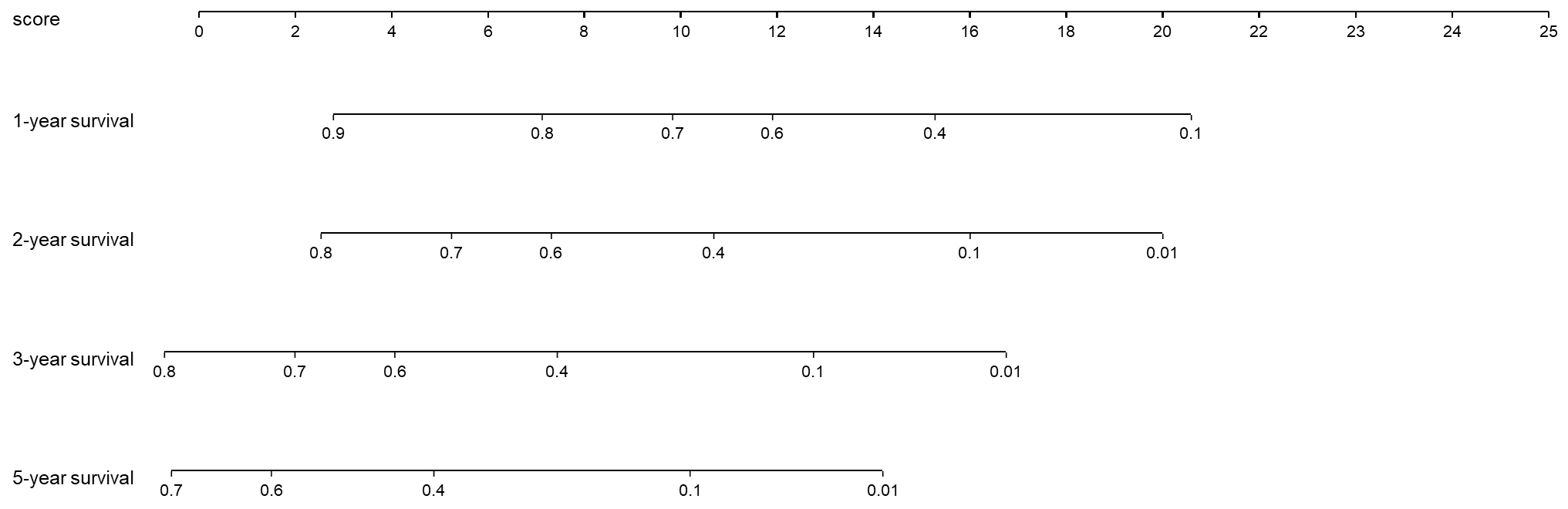


Supplementary figure 5 Survival probability map corresponding to each score.
